# Hemodynamic effects of sex and handedness on the Wisconsin Card Sorting Test: the contradiction between neuroimaging and behavioural results

**DOI:** 10.7717/peerj.5890

**Published:** 2018-11-21

**Authors:** Sigita Cinciute, Algis Daktariunas, Osvaldas Ruksenas

**Affiliations:** Institute of Biosciences, Life Sciences Center, Vilnius University, Vilnius, Lithuania

**Keywords:** Sex, Prefrontal cortex, Handedness, WCST, fNIRS, Gender, Hemodynamics, Neurovasculature, Near-infrared spectroscopy

## Abstract

This study investigated the potential role of sex and handedness on the performance of a computerised Wisconsin Card Sorting Test (WCST) in healthy participants by applying functional near-infrared spectroscopy (fNIRS). We demonstrated significant (*p* < 0.05) sex-related differences of hemodynamic response in the prefrontal cortex of 70 healthy participants (female, *n* = 35 and male, *n* = 35; right-handed, *n* = 40 and left-handed, *n* = 30). In contrast, behavioural results of the WCST do not show sex bias, which is consistent with previous literature. Because of this, we compared ours and sparse previous fNIRS studies on the WCST. We propose that, according to recent studies of neurovascular coupling, this contradiction between neuroimaging and behavioural results may be explained by normal variability in neurovascular dynamics.

## Introduction

Modern functional neuroimaging methods cover broad spatial and temporal scales (Pouratian et al., 2003) and facilitate *important* exploration of the human brain’s functional organisation in health and disease (Liu et al., 2015). Numerous statistical or methodological challenges are addressed with this complexity. However, some threats arise from fundamental conceptual challenges that remain widely underappreciated within the clinical and neuroimaging communities. First, the difficulty of isolating cognitive functions, and second, the difficulty in establishing specific mappings between the brain and human behaviour (Poldrack & Yarkoni, 2016).

An example of these problems could be the Wisconsin Card Sorting Test (WCST). It is a sophisticated test, which for more than four decades has been one of the most widely used cognitive tests of the prefrontal executive function (Nyhus & Barceló, 2009; Eling, Derckx & Maes, 2008; Monchi et al., 2001). However, clinical research and recent brain imaging have brought into question the validity and specificity of this test as a marker of frontal dysfunction (Nyhus & Barceló, 2009). One of the main reasons is a widespread activation in neural networks across multiple cortical regions during various stages of WCST performance and inconsistency of neuroimaging results despite the focus on healthy participants (Nyhus & Barceló, 2009). Most investigations on WCST performance in normal participants report a significant increase in metabolic or neural activity within frontal or prefrontal cortical regions, mainly the dorsal prefrontal cortex (dPFC) and ventrolateral prefrontal cortex (vPFC) (Nyhus & Barceló, 2009). To assess brain damage or disorder effects, functional neuroimaging studies contrast between patients and healthy controls populations (age and gender matched). For a long time, healthy participants were thought to be more or less homogeneous on WCST performance as no sex difference was identified (Nyhus & Barceló, 2009; Eling, Derckx & Maes, 2008). Thus, reported inconsistencies of neuroimaging results in healthy participants (Nyhus & Barceló, 2009) raise questions of whether the WCST fails to isolate frontal cognitive functions (thus contaminating neuroimaging data with various cognitive sub-processes), or functional brain mappings are sensitive to some aspects of neurophysiology which may not be related with behavioural results. Recent neuroimaging research demonstrated that individual variability should be thoroughly addressed before population-level inferences (Finn et al., 2015; Friedman & Miyake, 2017; Bidula et al., 2017). Usually, researchers attempt to reduce the impact of individual variability by controlling some critical factors for a specific study.

One of the standard factors of neuropsychological studies is the sex composition of participants. Sex differences have been of great interest in a variety of fields. The main two reasons in cognitive studies are (i) a known fact that gonadal hormones affect some functional cerebral asymmetries (Hausmann, 2016; Schöning et al., 2007; Amin et al., 2006), and (ii) evident sex-related differences in some cognitive tasks or overall information processing strategies (Bidula et al., 2017; Hill, Laird & Robinson, 2014; Zilles et al., 2016; McCarthy et al., 2012; Schmitz et al., 2017). Unfortunately, this shared knowledge is often used in favour of the researcher’s goals: that is to support insufficient experimental design based on male participants (resulting in sex bias when the effects from male reports are generalised for females (McCarthy et al., 2012; Check Hayden, 2010)), or to strengthen research conclusions according to the majority of research being focused on expectancy to detect sex differences in relation to test performance (Hill, Laird & Robinson, 2014; Hirnstein, Coloma Andrews & Hausmann, 2014; Miller & Halpern, 2014). From this perspective, cognitive and behavioural studies rarely investigate further, whether the obtained differences are due to underlying different cognitive processes, or due to the characteristics of neurophysiologic signal transduction. Recent interdisciplinary findings suggest that coupling between brain electrical activity, metabolism, and the empirically observed hemodynamic response is incredibly complex and functional neuroimaging oversimplifies the vascular response being induced by neural activity in a linear fashion (Wager et al., 2005; Attwell & Iadecola, 2002; Mishra et al., 2016; Huneau, Benali & Chabriat, 2015). Considering this, it is plausible that some inconsistencies between neuroimaging studies, as well as the contradiction between behavioural and neuroimaging results in some investigations, may be due to neural and vascular response mismatch, rather than because of underlying different cognition processes. Some recent scientific findings in animal (Lindauer et al., 2010) and explanatory molecular human biology models (Sweeney, Ayyadurai & Zlokovic, 2016) also suggest it. However, these studies investigate the pathophysiological changes of neurovascular coupling, while the basic mechanisms of neurovascular coupling remain unclear (Lindauer et al., 2010).

Another commonly controlling factor is handedness, mostly because of (i) sex-related odd ratios for left-handers (Papadatou-Pastou et al., 2008); (ii) and the unexplained phenomenon of collateralisation of preferred hand motor control in the left hemisphere together with language centres (for more than 90% of humans) (Hervé et al., 2013; Knecht et al., 2000). Both functions, preferred hand control and language, are located in the frontal cortex, and can plausibly impact the neuroimaging results of tests involving higher executive functions (Hervé et al., 2013; Habib, Nyberg & Tulving, 2003; Badre & D’Esposito, 2009). According to the Papadatou-Pastou et al. (2008) meta-analysis, men are more likely to be strong dominant left-handers than women, while only one subject out of 10 is a left-hander, and only one subject out of 10 left-handers is a woman (Papadatou-Pastou et al., 2008). Moreover, familial sinistrality (Papadatou-Pastou et al., 2008; Hervé et al., 2013; Mellet et al., 2014) together with the degree of handedness has been shown to be a promising measure of brain laterality in cognitive studies (Hervé et al., 2013; Corballis, 2014; Vuoksimaa et al., 2009). On the other hand, although specific gene products were proved to mediate the development of brain and body asymmetry, the real genetic role thereof in hand preference remains ambiguous (Corballis, 2014; Robinson et al., 2016; Brandler & Paracchini, 2014; Schmitz et al., 2017). As a result, right-handed bias in neuroscience research appeared, when some studies a priori, or a posteriori exclude left-handers from their analysis after observing some inconsistencies (Willems et al., 2014). Left-handed participants represent a substantial portion of the human population, and therefore left-handedness falls within the normal range of human diversity; thus, it is critical to account for this variation in our understanding of brain functioning (Schmitz et al., 2017; Willems et al., 2014).

Modern functional neuroimaging techniques, such as functional magnetic resonance imaging (fMRI), are often used to describe changes in brain activation during WCST performance (Nyhus & Barceló, 2009; Monchi et al., 2001; Alvarez & Emory, 2006). At this point, only very few studies have used functional near-infrared spectroscopy (fNIRS) for WCST (Fallgatter & Strik, 1998; Hashimoto, Uruma & Abo, 2007; Sumitani et al., 2006). We chose to conduct fNIRS as it is a very practical neuroimaging method for the extensive non-invasive measurement of hemodynamic responses. Moreover, the blood-oxygen-level-dependent (BOLD) signal detected in fMRI (Ogawa et al., 1990), and fNIRS haemoglobin concentration measurements (Jöbsis, 1977), despite their data acquisition differences, are both based on a common underlying phenomenon termed neurovascular coupling (Huneau, Benali & Chabriat, 2015; Phillips et al., 2016; Hillman, 2014). Neurovascular coupling is the process by which active brain regions induce a local increase in blood supply to match their energy demands via the dilation of arterioles through various signalling paths (Leybaert, 2005). Capillary dilation generates a significant portion of the blood flow increase evoked by neuronal activity (Hall et al., 2014) and is expected to contribute substantially to the observed hemodynamic response (Lindauer et al., 2010). Despite its importance and broad applicability, our understanding of neurovascular coupling in humans is still incomplete due to the lack of appropriate and consistent analysis strategies, and stimulation paradigms (Phillips et al., 2016; Hillman, 2014). There are relatively accepted overall sex differences in brain volume and morphology (Ruigrok et al., 2014; Lenroot & Giedd, 2010; Allen, Damasio & Grabowski, 2002) and grey/white matter ratio (Gur et al., 1999). Although possible differences in some aspects of the local capillary blood bed are still poorly addressed (Mishra et al., 2016; Walsh, 2016). Cerebral blood flow, volume and the metabolic rate of oxygen consumption are among the most often discussed parameters as they contribute to the biophysical model of BOLD (Kim & Ogawa, 2012). Regional cerebral blood flow has been shown to be influenced by sex and handedness previously (Rodriguez et al., 1988; Gur et al., 1982). Also, the more recent meta-analysis in a healthy and a large clinical psychiatric population support previous sex-related differences, and shows brain perfusion being notably increased in the prefrontal cortex (PFC) of females (Amen et al., 2017). However, the biophysical model implemented in fNIRS includes other important neurovascular dynamic factors, such as cerebral blood volume (CBV) and oxygenation (OXY) (Caldwell et al., 2016; Banaji et al., 2008) as well as some component of cellular level hemodynamic regulation via several different metabolic pathways (Hillman, 2014; Hamel, 2006; Filosa et al., 2016; Attwell et al., 2010; Ances et al., 2009; Walsh, 2016). In recent years, a substantial amount of research has been published supporting the ability of astrocytes to modulate vascular tone (Filosa et al., 2016; Ayata & Lauritzen, 2015; Bazargani & Attwell, 2016; Gratton, Chiarelli & Fabiani, 2017). Although auto-regulatory processes of the overall brain tissue blood supply, in general, might be indifferent or partially determined (Rodriguez et al., 1988; Gur et al., 1982; Amen et al., 2017), the microcirculation at the level of the penetrating arterioles, and capillaries is less well understood and controversial (Mishra et al., 2016; Hall et al., 2014). For example using in vivo animal models (rat and mice), it was shown that when the sensory input increases blood flow, capillaries dilate before arterioles and are estimated to produce 84% of the blood flow increase, while previously it was thought that capillaries usually do not significantly contribute (Hall et al., 2014). From this perspective, the examination of the potential direct and indirect sex hormone-related effects on between-subject variability in hemodynamic response is pivotal. A significant portion of brain maps are assumed to demonstrate different neural activity and thus cognition, while it may partly be the astroglial network (Giaume et al., 2010; Scemes & Spray, 2003) or other non-neural origin cells (Sweeney, Ayyadurai & Zlokovic, 2016; Hamel, 2006; Brooks, 2009) that induced normal neurovascular dynamics variability in response to neural activity.

Nonetheless, psychophysiological human studies are mostly relying on using non-invasive approaches, where new micro scale neuroscientific findings can be tested only as physiological and behavioural hypotheses. This study aims to examine the potential effects of sex and handedness on the WCST in large, consistently handed healthy subject groups using fNIRS. Surprisingly, only a few studies have used fNIRS for WCST and show inconsistent findings (Fallgatter & Strik, 1998; Hashimoto, Uruma & Abo, 2007; Sumitani et al., 2006). None of them proposed reasonable neurophysiology or cognitive strategies based on the explanation of their findings. Moreover, in all cases, studies were carried out in small or possibly heterogeneous groups of patients and healthy participants. To be consistent, we will focus only on healthy participants decomposed by sex and handedness. Moreover, the fNIRS community still does not have unified recommendations on how fNIRS data should be processed. Bearing this in mind, this study intentionally avoids the overuse of complicated data cleaning, processing and analysis procedures to ensure that the final results are not due to the interference of these factors, and only corresponds to the obtained signal. A few such interference examples, such as the introduction of systematic bias during preprocessing have been shown in other neuroimaging modalities (Hallquist, Hwang & Luna, 2013; Acunzo, MacKenzie & Van Rossum, 2012).

## Materials and Methods

### Participants

We recruited 98 healthy volunteers (female, *n* = 52; male, *n* = 46; right-handed, *n* = 51; left-handed, *n* = 40; and ambidextrous, *n* = 7) from a university using poster advertising. Informed written consent was obtained from all participants before inclusion, and the local ethics committee approved the study protocol. We excluded two participants with a lifetime history of neurological and cardiovascular pathology; 11 women with irregular menstrual cycles, and five using hormonal contraception (Warren et al., 2014); 10 participants whose optical spectroscopy recordings had more than four unreliable optode recordings (with saturated signal, or major movement artefacts); and seven ambidextrous participants (female, *n* = 4) were assessed with the Flinders *Handedness* Survey (Supplemental Material) (Nicholls et al., 2013). Left-handed participants reported no forced suppression of the preferred left-hand use. Using these strict criteria, we analysed data from 70 participants (female/male, *n* = 35/35; right-handed/left-handed, *n* = 40/30), (Table 1). Participants had an average age (±SD) of 21.74 ± 2.91 years, with no significant differences between men and women: 21.79 ± 3.83 and 21.71 ± 2.17 years, respectively.

**Table 1:** The composition of participant groups by sex and handedness.

Sex	Women		Men	
Handedness	Right	Left	Right	Left
Number of participants	20	15	20	15
Age, mean ± SD	21.45 ± 0.89	21.93 ± 3.49	20.55 ± 2.30	22.08 ± 3.56
FLANDERS score, mean ± SD	9.55 ± 0.89	−9.73 ± 0.80	9.65 ± 0.93	−8.79 ± 1.92

### Computerised WCST

The WCST was administered to all participants using the freely accessible digital WCST (v0.6) from the Psychology experiment building language (PEBL v0.13) test battery (Mueller & Piper, 2014). Oral and written instructions for the experiment and WCST were provided: participants were requested to use their performance feedback to find the correct classification principle by trial and error for 128 cards with geometric figures that vary according to three perceptual dimensions (colour, symbol, or number). There was no time limit to finish the test. The whole experiment was recorded in a single individual session. This study investigated the overall effect of WCST on the hemodynamic responses of the PFC. Due to the nature of the WCST and a low sampling rate of fNIR400, we were not able to further investigate aspects of hemodynamic response during category shifting.

### fNIRS recording system

The study was conducted using a continuous type 16 optodes (up to 48 channels)^1^1Optode is a term in spectroscopy to name a pair of constituted by emitter and a photodetector. As multiple wavelengths may be used in the emitter, one optode may form from two to several channels. device fNIR400 from Biopac, USA. The sensor is designed to monitor rostral cortical areas beneath the forehead, including Brodmann areas 10, 9, 45, and 46 (Ayaz et al., 2010; Izzetoglu et al., 2007). It has a 2.5 cm source-detector separation allowing for approximately 1.25 cm penetration in depth and a temporal resolution of two Hz. Measurement values are in micromolar units (μMol/L), in contrast with a pre-recorded 10 s baseline. The probe was aligned with the electrode positions FP1 and FP2 based on the international 10–20 EEG system (Ayaz et al., 2006). For optical data acquisition, we used Cognitive Optical Brain Imaging Studio software (v1.3), (Drexel University).

### Preparation of fNIRS data

The fNIRS signals were pre-processed as described: (a) a visual inspection of raw data quality was performed; (b) a low-pass finite impulse response filter (order 20) with the cut-off frequency set to 0.1 Hz (Izzetoglu et al., 2005) was used to eliminate respiration and heart pulsation artefacts; (c) the sliding-window motion artefact rejection algorithm was used (window size 10, threshold range from 3 to 25, (Ayaz et al., 2010)); (d) a modified Beer–Lambert Law (Cope & Delpy, 1988) was implemented to calculate the following hemodynamic parameters: change in the oxygenated haemoglobin (HbO_2_), deoxygenated haemoglobin (HbR), total blood volume (HbT, the sum of HbO_2_ and HbR), and OXY (the difference between HbO_2_ and HbR). Data pre-processing, calculation of the hemodynamic response parameters and topographic representations were performed using fnirSoft (Ayaz, 2010) (version 3.5, fNIR Devices, Potomac, MD, USA).

### Statistical data analysis

#### Wisconsin Card Sorting Test

Two-way analysis of variance (ANOVA) were used to examine the sex and handedness effect on the number of perseveration errors, correct test sorting rate and total duration of WCST performance.

#### fNIRS data

To explore the effect of sex and handedness on the hemodynamic response parameters (HbO_2_, HbR, HbT, and OXY) in 16 optodes, we conducted two-way repeated measures multivariate analysis of variance (RM-MANOVA). Tests were conducted with two pairs of hemodynamic response measures (‘HbO_2_’ and ‘HbR’; ‘HbT’ and ‘OXY’), as HbT and OXY are linear combinations of HbO_2_ and HbR and were highly correlated (*r* > 0.8). Therefore, these pairs were examined separately. The within-subject factor was defined as ‘Optode’ with sixteen dependent variables per measure. The alpha level was set at 0.05. Wilks’s lambdas were used to test whether there are differences between the means of independent groups of participants on a combination of dependent variables. Effect sizes were examined as the proportion of variance accounted for by partial η^2^. Whenever the sphericity assumption was violated, Greenhouse-Geisser corrected values were used for further analysis. Tests were carried out with Bonferroni adjustments (α = 0.05) for multiple comparisons correction where necessary.

Concerning the question which optodes are significantly different among the discrete groups of participants, a one-way univariate analysis of variance (ANOVA) was conducted. A total of 16 dependent variables were analysed by a single independent factor, ‘Group,’ (right-handed females, left-handed females, right-handed males, and left-handed males; see Table 1). A multiple comparisons correction was carried out with Bonferroni adjustments (α = 0.05).

Statistical analyses were conducted with SPSS (version 20.0; SPSS Inc., Chicago, IL, USA).

## Results

### Wisconsin Card Sorting Test

Consistent with previously reported findings, we did not observe any statistically significant sex or handedness bias on WCST performance (Nyhus & Barceló, 2009; Eling, Derckx & Maes, 2008). The mean number of perseveration errors was 14.45 ± 6.50 (99% CI [12.72, 16.18]), and the mean correct sorting rate was 82.08 ± 7.78% (99% CI [80.02, 84.14]). No significant differences were found comparing by sex or handedness. The average duration of the WCST for all participants was 268.74 ± 60.88 sec (99% CI [252.87, 284.61]). We did not observe any significant differences in the duration of task performance regarding sex and handedness.

### fNIRS data

#### The sex and handedness effects on the hemodynamic response

The results of the two-way RM-MANOVA tests revealed a statistically significant main effect of sex (*F* (2, 65) = 3.521, *p* = 0.035; Wilk’s Λ = 0.902, η^2^ = 0.098). A non-significant effect of handedness (*F* (2, 65) = 2.977, *p* = 0.058; Wilk’s Λ = 0.916, η^2^ = 0.084) was observed. The interaction of these two factors was not significant (*F* (2, 65) = 2.271, *p* = 0.111; Wilk’s Λ = 0.935, η^2^ = 0.065).

The strongest statistically significant univariate effect was obtained considering sex and the HbO_2_ measure (*F* (1, 66) = 7.146, *p* = 0.009, η^2^ = 0.098), (Table 2). A moderately significant effect of handedness was obtained on HbR (please see Table 2 for details). Hemodynamic response derivatives such as HbT and OXY were impacted differently: sex as a factor (*p* = 0.043) had a weaker effect than handedness (*p* = 0.022) on the total haemoglobin; while OXY (difference between neurovascular supply and demand) showed to be significantly affected by sex (*p* = 0.042), (please see Table 2 for details), and more interestingly, it showed a sex by handedness interaction, (*F* (1, 66) = 4.564, *p* = 0.036, η^2^ = 0.065).

**Table 2:** Significant effects of sex and handedness on different hemodynamic parameters (*N* = 70, α = 0.05) from two-way RM-MANOVA tests.

Dependent variables	Independent variables	
	Sex	Handedness
HbO_2_	***F* (1, 66) = 7.146, *p* = 0.009, η^2^ = 0.098**	*F* (1, 66) = 1.942, *p* = 0.168, η^2^ = 0.029
HbR	*F* (1, 66) = 0.003, *p* = 0.958, η^2^ = 0.000	***F* (1, 66) = 4.338, *p* = 0.041, η^2^ = 0.062**
HbT	***F* (1, 66) = 4.244, *p* = 0.043, η^2^ = 0.060**	***F* (1, 66) = 5.519, *p* = 0.022, η^2^ = 0.077**
OXY	***F* (1, 66) = 4.322, *p* = 0.042, η^2^ = 0.061**	*F* (1, 66) = 0.006, *p* = 0.807, η^2^ = 0.001

**Note:**

HbO_2_, HbR, and HbT, OXY pairs were examined separately. No Significant values are bold. Only one statistically significant interaction of sex and handedness was obtained, and it was regarding oxygenation (OXY), (*F* (1, 66) = 4.564, *p* = 0.036, η^2^ = 0.065).

**Figure 1:**
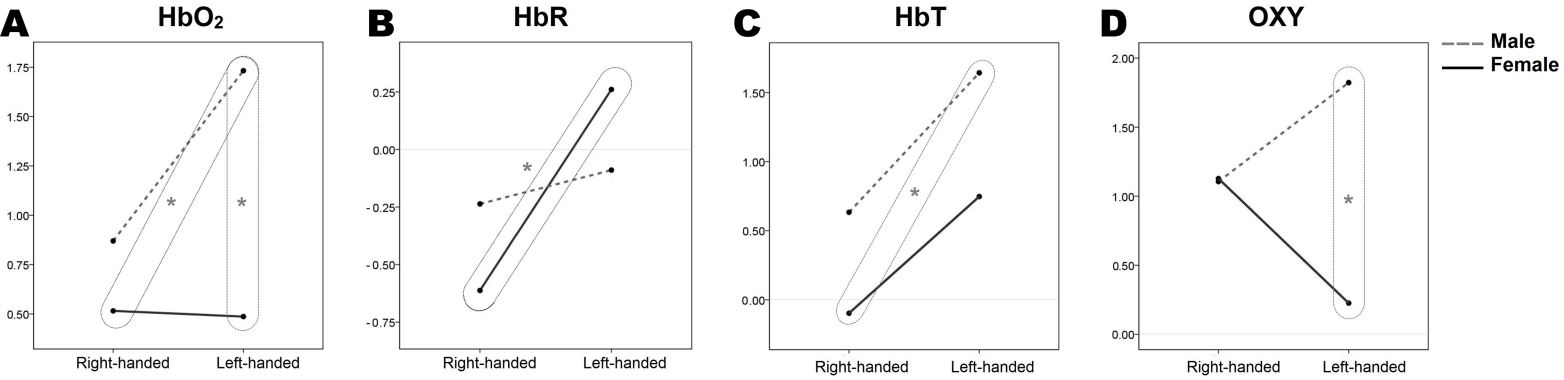
Estimated marginal means of hemodynamic response parameters (A, HbO_2_; B, HbR; C, HbT; D, OX) regarding sex and handedness. Box and the star indicate significant differences between groups, (*p* < 0.05). Results are from 70 participants’ two-way RM-MANOVA tests (female/male, *n* = 35/35; right-handed/left-handed, *n* = 40/30; subsequently right-handed female/male, *n* = 20/20, and left-handed female/male, *n* = 15/15).

The statistical summary of between-subject effects can be found in Table 2. The mean response for each factor, adjusted for all variables in the models, is shown in Fig. 1 as estimated marginal means.

#### The significant differences between the groups of participants

Independent sample *t*-tests were conducted for the further investigation of differences between the means of independent groups of participants (Table 1) for each hemodynamic response parameter. Significantly different groups of participants are additionally marked in Fig. 1.

Comparing hemodynamic parameters, two sample *t*-tests revealed that the HbO_2_ concentrations of left-handed males (1.73 ± 0.29) differ significantly from right-handed (0.51 ± 0.27) and left-handed (0.48 ± 0.25) females, (*t* (33) = −3.039, *p* < 0.05) and (*t* (28) = −3.230, *p* < 0.05) accordingly. Also, males show the tendency of having higher overall measured HbO_2_ concentrations than females (Fig. 1). Further, statistically significant HbR concentrations differences were within females, comparing right-handers (−0.61 ± 0.17) and left-handers (0.26 ± 0.24), (*t* (33) = 3.045, *p* < 0.05). Examining the differences in total haemoglobin increase between the groups of participants it was found that right-handed females (−0.10 ± 0.24) and left-handed males (1.63 ± 0.42) are significantly different (*t* (33) = 3.807, *p* < 0.05), whereas left-handed females and right-handed males were very similar (see Fig. 1, where *y*-axis values for corresponding groups are at the same level). For the OXY parameter, a statistically significant concentration difference was found for left-handed females (0.22 ± 0.33) and males (1.82 ± 0.42), (*t* (28) = −2.994, *p* < 0.05), confirming the previously found interaction of sex and handedness in the two-way RM-MANOVA test, (*F* (1, 66) = 4.564, *p* = 0.036, η^2^ = 0.065). As mentioned before, OXY is the difference between HbO_2_ and HbR concentrations, or how much of cerebrovascular overcompensation (the difference between the supply and demand) was present. Right-handed females and males were equivalent (see Fig. 1, where OXY plot *y*-axis values for right-handed groups match), and left-handed females and males were significantly different in overall oxygen consumption during the WCST.

#### Sex effects on the HbO_2_ concentration

**Figure 2:**
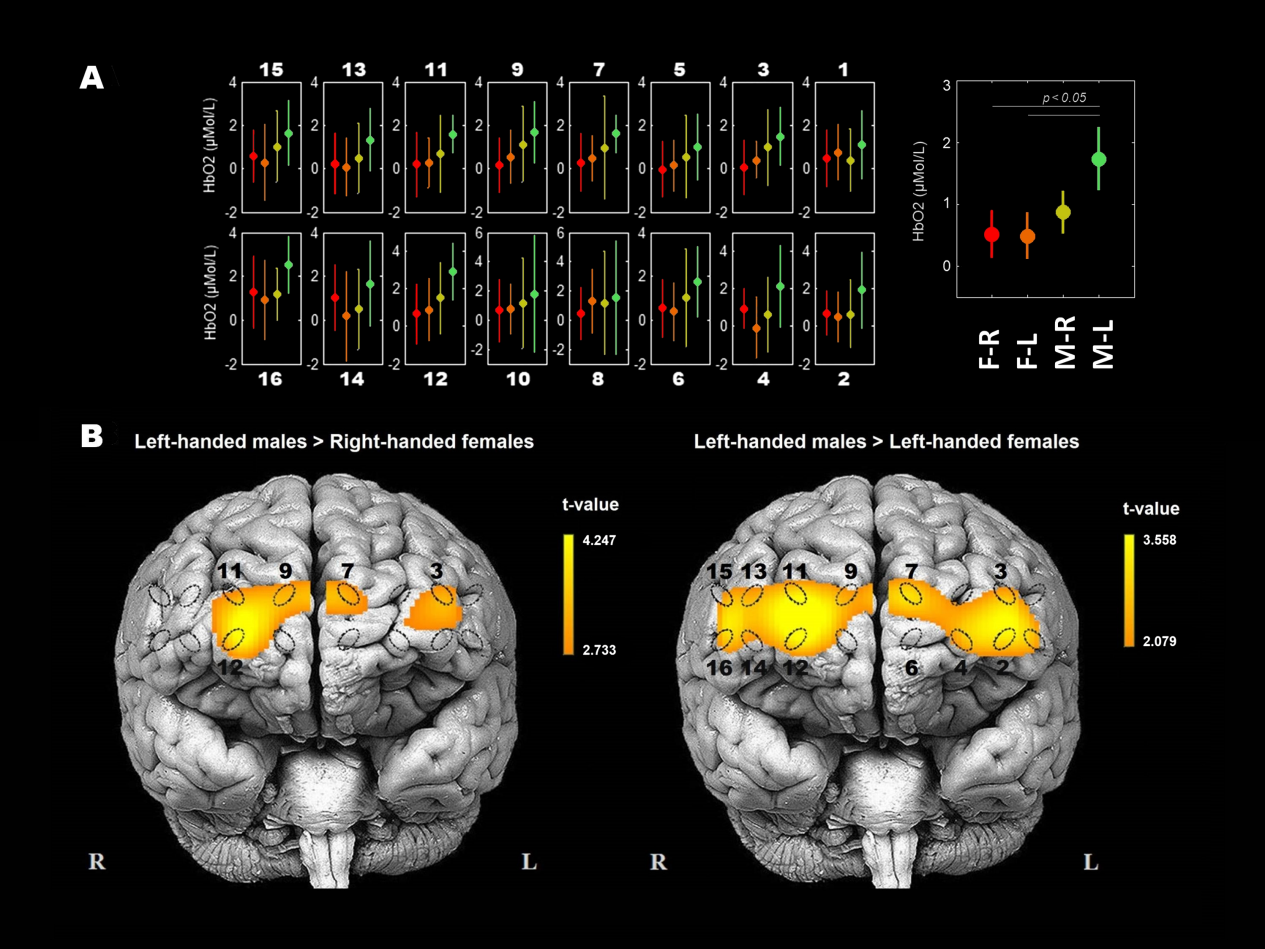
Spatial differences of HbO_2_ concentration between groups of participants during the WCST. (A) Error plot of HbO_2_ mean (±SD) concentrations of HbO_2_ between participants’ groups in each of 16 optodes, and the mean (±SD) concentrations of HbO_2_ across optodes per group. Participants groups are: female right-handed, *n* = 20, F-R; female left-handed, *n* = 15, F-L; male right-handed, *n* = 20, M-R; male left-handed, *n* = 15, M-L). (B) Statistical activation maps (*t*-values, *p* < 0.05) display where the obtained HbO_2_ concentrations were significantly higher for left-handed males than right-handed females (*t* (33) = 3.039, *p* < 0.05) and left-handed females (*t* (28) = 3.230, *p* < 0.05). Significant optodes are outlined. Statistical values were spatially visualised using the BSpline18 interpolations method. The measured areas are marked with ellipses.

The strongest statistically significant simple main effect was obtained considering sex and HbO_2_ concentrations (*p* = 0.009; see Table 2). Males in general show the tendency of having higher overall measured HbO_2_ concentrations than females and left-handed males differ significantly from both groups of females (Fig. 1). Independent two-sample *t*-tests were used to demonstrate spatial differences of HbO_2_ concentration between groups of participants (Fig. 2): the error plots in Fig. 2A show the distribution of mean (±SD) concentrations of HbO_2_ in 16 optodes (left), and the mean (±SD) across optodes per group (right). The variability of means between groups is apparent and consistent (from left to right: right-handed females, left-handed females, right-handed males, and left-handed males; also see Table 1). The statistical activation maps (*t*-values, *p* < 0.05) in Fig. 2B show where HbO_2_ was significantly higher for left-handed males than right-handed or left-handed females. The substantial differences between these three participants groups for HbO_2_ are obtained in the right superior frontal gyrus (9–12th optodes) and the left middle frontal gyrus (2–4th optodes).

#### The interaction of sex and handedness regarding oxygenation

**Figure 3:**
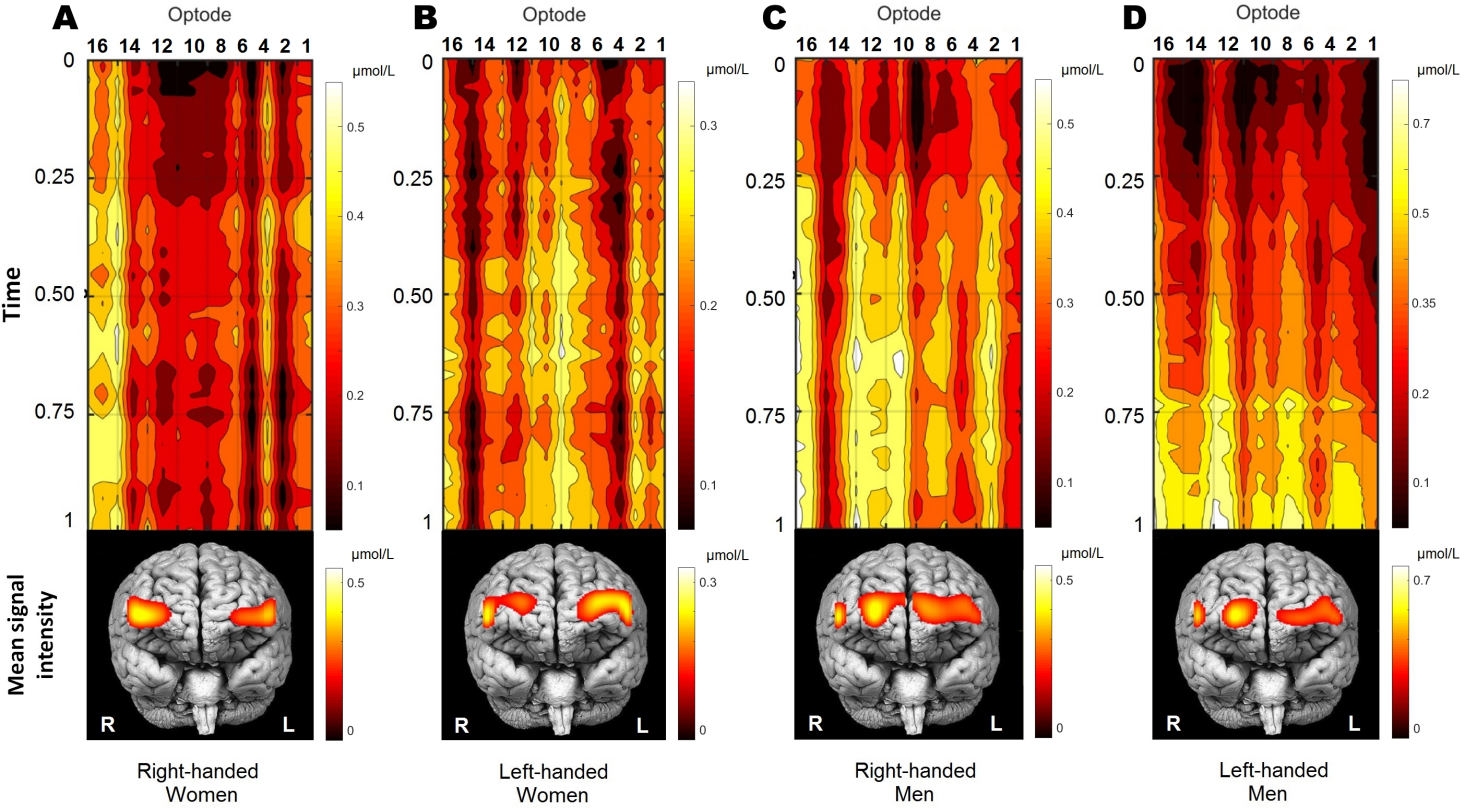
The distribution of oxygenation for participants’ groups (A, right-handed women; B, left-handed women; C, right-handed men; D, left-handed men) during the performance of WCST. Visualisation of time-series oxygenation changes, together with mean spatial signal distribution on frontal brain view. All 70 participants were decomposed into four groups by gender and handedness. Time-series of OXY concentrations is normalised by the max value. The mean signal is shown below. Recordings were also normalised in time (from the start of WCST being 0, and to the end of WCST being 1) individually and later averaged per group. Note that time series presentation does not correspond directly to the frontal brain view distribution below due to double line probe geometry. Values were spatially visualised with BSpline18 interpolations method.

The OXY may be interpreted as a measure of how much of cerebrovascular overcompensation, or the difference between supply and demand, is present at the particular brain area. At this point, the statistically significant interaction of sex and handedness on OXY was found, (*p* = 0.036; see Table 2). The measure of OXY is not often used, as it does not directly correspond to current biophysical models of fNIRS or fMRI, and is considered more as an additional measure. However, its physiological interpretation may give some insights into cerebrovascular regulation: the cerebrovascular reactivity (active vasodilatation of neurovasculature to increase oxygen supply) and relative metabolic rates of oxygen consumption (corresponding to the brain tissue level rather than cellular metabolism) may demonstrate significantly different patterns. Considering previous findings, right-handed females and males are very similar (see Fig. 1), and left-handed females and males are significantly different, (*t* (28) = −2.994, *p* < 0.05). The significant interaction can be subsequently explained by the significant effects of sex and handedness on HbO_2_ and HbR, as the OXY is a difference between these two parameters. Nevertheless, 2D contour plots were created to demonstrate the spatial and temporal distribution of OXY change during the WCST (Fig. 3).

Figure 3 shows qualitative and quantitative OXY distribution for all four participants’ groups during the performance of WCST. Because the duration of the WCST performance was individual (see section “Wisconsin Card Sorting Test”), each participant’s data was normalised in time and amplitude before the group average. Normalisation in time was made from the start of the WCST (0), and to the end of the WCST (1) individually. To cancel out signal drift and scale the amplitude, each subject was normalised by its maximum value:

$$X = {{{x_i}} \over {\left| {\max {x_i}} \right|}};$$

where *x_i_* is a signal and max *x_i_* is the maximal value of a signal obtained in optode number *i*. Participants performed the WCST at their own pace before group analysis data were first rescaled in time into a range of [0,1].

As a result, the temporal dynamics of OXY changes could be defined as quite steady during the WCST performance in all groups, except for left-handed men, who reach their peak activity in the last quarter of the experiment. Frontal brain views in the lower part of Fig. 3 show the localisation of the within-group mean signal intensities, which is quite distinctive between groups. To sum up, OXY differences between participants regarding their sex and handedness demonstrate the cerebrovascular reactivity and relative metabolic rates of oxygen consumption creating significantly different patterns.

## Discussion

The current study evaluated whether two common biological factors, namely sex and handedness, have an impact on the neuroimaging results for PFC functioning during the WCST. Analyses revealed significant sex-related hemodynamic response differences among participants in HbO_2_ concentrations, where males demonstrate higher overall measured HbO_2_, concentrations than females. Meanwhile, the effect of handedness remains ambiguous: for the chosen alpha level, it was not statistically significant at the multivariate level, but the moderately significant effect of handedness was obtained on HbR alone (Fig. 1; Table 2). More interestingly, a statistically significant interaction of sex and handedness was obtained on OXY (Fig. 1; Table 2). At the same time, no sex or handedness effects were apparent on the behavioural level of the WCST performance (see section “Wisconsin Card Sorting Test” for more details). These findings partially support the hypothesis that some functional brain maps of the WCST may be sensitive to neurophysiological heterogeneity which is not apparent or necessarily related to the behaviour. At the same time, it cannot exclude that the WCST fails to isolate frontal cognitive functions fully, and visible heterogeneity in neuroimaging is due to cognitive sub-processes which are merely not caught by WCST behavioural results. Nevertheless, our results provide some new insights into this problem.

By this study we addressed the high complexity of reasoning in similar neuropsychological, cognitive studies: numerous methodological, technical and statistical challenges have to be addressed in advance regarding sex and handedness contribution. Unfortunately, this may lead to the inaccurate causal inference, subject selection bias, and overlook of some fundamental questions such as the neurophysiological background of results in question. For example, it has been shown that brain perfusion or cerebral blood flow (CBF) is notably increased in the PFC of females (Amen et al., 2017). However, according to our study results, males demonstrate higher overall measured HbO_2_ concentrations than females. A few fNIRS studies on other cognitive tasks also found the amplitudes of HbO_2_ being significantly higher in males than females (Li, Luo & Gong, 2010; Yang et al., 2009; Jaušovec & Jaušovec, 2010). These findings do not necessarily contradict each other, as perfusion is defined by CBF, where HbO_2_ is related to CBF and CBV. Cerebral blood flow is maintained by a decrease in the resistance of the distal vasculature, where as a result, both CBV and the CBV/CBF ratio would increase, while the extraction of oxygen from the circulating blood may remain the same (Powers, 1991). In other words, cerebrovascular regulation is a dynamic process, and different neuroimaging methods such as fNIRS, fMRI, positron emission tomography (PET), and photon emission computed tomography (SPECT) which are based on different biophysical models of the same phenomenon can provide different data. As was briefly summarised in the introduction, despite its importance, the understanding of neurovascular coupling is still incomplete. At this point, implementation of more recent cellular level findings and computational modelling implementation on existing paradigms is highly favourable (Hillman, 2014; Sweeney, Walker-Samuel & Shipley, 2018).

Further, only a few previous WCST studies were performed with fNIRS. Fallgatter & Strik (1998) and Hashimoto, Uruma & Abo (2007) had a low spatial resolution (two optodes) or one region of interest per hemisphere. Their conclusions are restricted to the right hemisphere being more active than the left in healthy controls. Another, Sumitani et al. (2006) study had a higher spatial resolution (24 optodes), and, similarly with the present study, showed patterns of multiple activities within prefrontal cortices. However, the fNIR400 system covers rostral and more lateral regions of the PFC. The ETG-400, used by Sumitani et al., covers rostral and more dorsal regions of the PFC. Thus, these study results are partially comparable to those of Sumitani et al. (2006) as probe projection onto the PFC differs, and no common spatial normalisation in fNIRS is available. Sumitani and colleagues propose different patterns of the activation in the PFC among healthy participants but do not provide consistent and reasonable neurophysiological or cognitive strategies based explanation of their findings. At this point, our study provides comprehensive hypotheses including all or nearly all elements or aspects of contradiction between behaviour and neuroimaging regarding WCST. Furthermore, our finding that some significantly different patterns of cerebrovascular dynamics between participants can be observed directly from minimally manipulated fNIRS data raise several important questions: (i) whether contradictions between neuroimaging and behaviour exist within other cognitive tests, particularly at those which usually do not show sex bias, and (ii) whether similar or contrasting findings were observed in resting state functional studies?

The first question can be illustrated with two fNIRS studies where sex-related differences in hemodynamic responses have been reported during verbal working memory (Li, Luo & Gong, 2010) and mental arithmetic (Yang et al., 2009) tasks. Both studies found the amplitudes of HbO_2_ being significantly higher in males than females. In both cases, to some extent it was interpreted as evidence of different information processing patterns, although sex-related behavioural differences were not found. Most likely because both tasks are presumably considered to demonstrate sex-related differences (Ruigrok et al., 2014; Lenroot & Giedd, 2010). Authors do not provide a clear explanation as to why the lack of correspondence between neuroimaging and behavioural results is considered as evidence of different cognition than the previously mentioned claim? As was proposed in the introduction, astroglial network (Giaume et al., 2010; Scemes & Spray, 2003) and other non-neural origin cells (Sweeney, Ayyadurai & Zlokovic, 2016; Hamel, 2006; Brooks, 2009) have been recently shown to have a considerable impact on normal neurovascular dynamics. By this, their conclusions are highly debatable, because the findings obtained with fNIRS and fMRI studies on nonlinear properties of hemodynamic response (Wager et al., 2005; Huneau, Benali & Chabriat, 2015; Fabiani et al., 2014), as well as the sex-related differences at rest (Jaušovec & Jaušovec, 2010; Bidula et al., 2017; Chuang & Sun, 2014) were not considered.

Regarding the second question, we were able to find one fNIRS-based resting state study, exploring gender-related differences in PFC activity in a total of 40 healthy right-handed participants (Chuang & Sun, 2014). This fNIRS resting-state functional connectivity study proposed that inferior PFC is significantly different between male and female groups with both time-series and spectrum analyses (Chuang & Sun, 2014). However, no overall haemoglobin concentration comparison was provided. Also, resting states of 300 healthy right-handed participants were assessed by fNIRS and EEG in a whole brain approach (Jaušovec & Jaušovec, 2010). This team proposed that males display a higher level of HbO_2_ than females (Jaušovec & Jaušovec, 2010), which is consistent with our findings. Furthermore, the authors indicated that males and females significantly differ in the default mode of brain activity considering both modalities (Jaušovec & Jaušovec, 2010). In contrast, no comparable resting state studies in healthy participants regarding gender differences were found with widely used fMRI, as overall BOLD signal intensities are not usually compared, and more sophisticated methods are proposed instead (Biswal, 2015; Lee, Smyser & Shimony, 2013). Summarising these contradictions, the question remains as to whether the discussed fNIRS findings imply different intrinsic neural activity or normal variability in neurovascular dynamics.

Despite important questions that have emerged in this study, some an equally important criticism needs to be addressed in future research. For example the possible contamination of fNIRS data that could alter some of the results cannot be entirely discarded. The fNIRS signal contamination from extra-cerebral tissues on overall hemodynamic measurements is hard to overcome, due to a path of light being unavoidably heterogeneous (Tachtsidis & Scholkmann, 2016). A few steps minimised the possible impact. First, all participants in this study were Caucasians, and acceptable signal levels for measurements were reached by individually adjusting light intensities before the experiment. Second, a tight fit of the probe to the head for squeezing out the scalp blood was made. Although without explicit control, extra-cerebral contamination cannot be entirely discarded as most of the fNIRS signal is thought to come from the brain surface where the cortical vasculature is dense (Reina-De La Torre, Rodriguez-Baeza & Sahuquillo-Barris, 1998). At this point, apparent differences between men and women cannot be explained solely by scalp-origin physiological confounds without empirical proof. Moreover, even if this would be the case, most of the commercially available continuous wave type fNIRS devices still do not have implemented solutions (Tachtsidis & Scholkmann, 2016). This illustrates the need for the fNIRS community to define some clear and consistent data processing guidelines to assure proper and widely accepted inference strategies. Another concern that has to be addressed is that no spatial or volumetric standardisation is currently implemented to discard natural sex differences in brain volume and morphology in fNIRS. Thus, the sex-related properties of the cerebral vasculature may be a confounding effect in some studies. For these reasons, this study intentionally avoids overuse of complicated data cleaning, processing and analysis procedures to assure that the final results are not due to any interference, and they only correspond to an obtained physiological signal; leaving more sophisticated approaches to be addressed after some consents are being met first.

Another limitation that should be kept in mind is that in this study, only the overall effect of the WCST on the hemodynamic responses of the PFC was investigated. Due to the nature of the WCST and a low sampling rate of fNIR400, we were not able to observe hemodynamic response during category shifting. This potentially reduces our understanding of sex and handedness-related interactions on the WCST. Also, the ambiguous effect of handedness may be explained by handedness being identified solely on a questionnaire. Therefore, more objective measurements might be used to associate it with functional brain lateralisation (Prichard, Propper & Christman, 2013; Hervé et al., 2013; Mellet et al., 2014). Moreover, a whole brain approach could give better insight, as frontal and non-frontal cortical activations during WCST could be subsequently addressed (Nyhus & Barceló, 2009). Despite the difficulties of addressing handedness effect on WCST, the sex-related odd ratios for left-handers cannot be neglected (Papadatou-Pastou et al., 2008). Likewise, the results of this study demonstrate females HbR differences regarding handedness, and distinctive spatial hemodynamic response distribution between left-handed men and women (Table 2; Figs. 1–2). Thus, it provides new data-based insights into the discussion about the efficiency of the WCST.

## Conclusions

The study demonstrates an evident sex effect on the neuroimaging results of the WCST in healthy participants, while no differences were obtained on the behavioural level. These findings are consistent with previous fNIRS neuroimaging and behavioural WCST results. Thus, we interpret this contradiction between neuroimaging and behavioural results as neurophysiological evidence for normal variability in neurovascular coupling dynamics. This claim is supported by the recent scientific findings in the regulation of neurovascular coupling and origin of hemodynamic response as well as other neuroimaging studies.

Also, this study highlights the difficulty of an accurate interpretation of a possible handedness impact on the neuroimaging results, as with current knowledge of this phenomena, it is not evident whether handedness-related functional brain lateralisation may profoundly interact with frontal cortical activation during WCST or not. Altogether, the results of this study suggest that handedness may explain some variability of WCST neuroimaging results. Together with subject selection bias across studies, which were available for comparison, we conclude that it may be fully revealed with a more sophisticated approach.

Finally, we summarise the high complexity of reasoning, regarding sex and handedness in similar neuropsychological and cognitive studies, as well as the main concerns associated with previous fNIRS studies on WCST, which are discussed as the overall ambiguity of neurovascular coupling.

## Supplemental Information

10.7717/peerj.5890/supp-1 Supplemental Information 1 Lithuanian version of Flanders hand survey.

## Additional Information and Declarations

### Data Availability

The following information was supplied regarding data availability: Cinciute, Sigita (2018): Cinciute et al. 2018 PeerJ. figshare. Collection. DOI 10.6084/m9.figshare.c.4277624.v1.
