# Supplementary material for: Hemodynamic effects of sex and handedness on the Wisconsin Card Sorting Test: the contradiction between neuroimaging and behavioural results
Source: PeerJ. 2018 Nov 21;6:e5890. doi: 10.7717/peerj.5890 (PMC6252064; doi:10.7717/peerj.5890)
Supplement: Supplemental Information 1 [file peerj-06-5890-s001.pdf]

The original English version: M. E. R. Nicholls et al., “The Flinders Handedness survey (FLANDERS): a brief measure of skilled hand preference.,” *Cortex*. **49**(10), 2914–2926, Elsevier Ltd (2013), [doi:10.1016/j.cortex.2013.02.002].

The first use of Lithuanian version: Genyte, V., Griskova-Bulanova, I. “Klausos nuostoviųjų atsakų lateralizacijos tyrimas: dešiniarankių ir kairiarankių palyginimas. Proceedings of the international conference *Virtual instruments in biomedicine*, 2015, p. 54–58. *Reused with the permission.*

Flinderso Rankiškumo Testas (FLANDERS)

Pavardė: …………………………………… Vardas: ……………………………………….

Gimimo data: ……………………………… Lytis (vyras/moteris) ………………………..

Jums bus pateikta dešimt klausimų apie tai, kurią ranką Jūs naudojate skirtingose situacijose. Prašome varnele pažymėti vieną atsakymo langelį, labiausiai nusakantį, ar tam tikrai užduočiai atlikti naudojate dešinę, kairę ar abi rankas vienodai. Atkreipkite dėmesį, kad atsakymą „abiem“ pasirinkite tik tuo atveju, kai nei viena iš rankų nėra pranašesnė už kitą. Prašome atsakyti į visus pateiktus klausimus, net jei su kažkokia užduotimi nesate susidūręs - tiesiog pasistenkite įsivaizduoti kaip galėtumėte ją atlikti, ir pažymėkite atsakymo variantą.

		Kaire	Abiem	Dešine
1.	Kuria ranka Jūs rašote?			
2.	Kuria ranka naudojate šaukštą, kai valgote?			
3.	Kurioje rankoje laikote dantų šepetėlį, kai valotės dantis?			
4.	Kurioje rankoje laikote degtuką, norėdami jį uždegti?			
5.	Kurioje rankoje laikote trintuką, kai trinate pieštuku padarytas klaidas?			
6.	Kurioje rankoje laikote adatą, kai siūnate?			
7.	Kai tepate sviestą ant riekės duonos, kurioje rankoje yra peilis?			
8.	Kuria ranka laikote plaktuką?			
9.	Kurioje rankoje laikote skustuką, kai lupate obuolį?			
10.	Kuria ranka piešiate?			

Rankiškumo balas *(prašome nepildyti):*	
